# The complexity of diagnosing sarcoma in a timely manner: perspectives of health professionals, patients, and carers in Australia

**DOI:** 10.1186/s12913-020-05532-8

**Published:** 2020-08-03

**Authors:** Rhys Weaver, Moira O’Connor, Richard Carey Smith, Georgia KB Halkett

**Affiliations:** 1 School of Nursing, Midwifery and Paramedicine, Curtin University, GPO Box U1987, Perth, WA 6845 Australia; 2 WA Cancer Prevention Research Unit (WACPRU), School of Psychology, Curtin University, Perth, Australia; 3 Department of Orthopaedic Surgery, Sir Charles Gairdner Hospital, Nedlands, WA Australia; 4 Perth Children’s Hospital, Perth, WA Australia; 5 Perth Orthopaedic and Sports Medicine Centre, Perth, WA Australia

**Keywords:** Sarcoma, Qualitative research, Interviews, Patients, Carers, Thematic analysis

## Abstract

**Background:**

Prolonged diagnosis intervals occur more often in rare cancers, such as sarcoma. Patients with a delayed diagnosis may require more radical surgery and have a reduced chance of survival. Previous research has focused on quantifying the time taken to achieve a diagnosis without exploring the reasons for potential delays. The aim of this study was to explore patients’, carers’, and health professionals’ perceived barriers to timely diagnosis and referral for treatment for sarcoma.

**Methods:**

Semi-structured interviews were conducted with: health professionals working with sarcoma (*n* = 21); patients who have been diagnosed with sarcoma (*n* = 22); and carers of patients diagnosed with sarcoma (*n* = 17). Interview transcripts were analysed using thematic analysis.

**Results:**

Four overarching themes were identified: patient perception of symptoms, difficulties of diagnosis, lack of experience, and availability of health services. Diagnosis was prolonged by the limited availability of health services, lack of prompt referrals to a sarcoma specialist centre, and diagnostic challenges. Intervals also occurred when patients underestimated the severity of their symptoms and did not seek prompt medical consultation.

**Conclusions:**

Patients with a potential sarcoma need to be promptly referred to a sarcoma specialist centre and additional diagnosis pathways need to be developed to reduce the rate of patients being referred to wrong specialists. Sarcoma education must be embedded in medical courses and professional development curricula. A public health approach should be taken to improve sarcoma knowledge and health seeking behaviours in the community.

## Background

Sarcoma is a group of very rare primary bone and soft tissue tumours accounting for less than 3% of all cancers [1]. Sarcomas can occur at any age, including in childhood and adolescents [2]. Sarcomas arise in connective tissues such as fat, cartilage and bone, and may occur in almost any anatomical location [3, 4]. In Australia approximately 1200 new cases are diagnosed each year, accounting for approximately 1% of all adult malignancies and 15% of paediatric malignancies [1]. Currently, two in five patients diagnosed with sarcoma will die from their disease [1]. The 5-year post-diagnosis sarcoma survival rate is estimated to be approximately 60% in Western countries (such as the UK, US and Australia), ranging from about 90% survivorship for low grade lesions, to less than 50% survivorship for high grade tumours [5]. Patients experience a variety of unmet needs, including access to information, health services, psychosocial support, and financial support [6]. Ideally, sarcoma patients are managed by a multidisciplinary sarcoma specialist team, which may include receiving clinical management and support from a sarcoma nurse specialist (Weaver et al.; manuscript under review).

### Importance of a timely diagnosis

A systematic review concluded that, for commonly occurring cancers, expedited diagnosis improves cancer outcomes [7]. For sarcoma patients, early diagnosis is essential to reduce the magnitude of surgery and increase survival chances [8]. Delays in diagnosis are associated with larger tumours, increased risk of metastases, and increased risk of amputation rather than limb salvage surgery [9]. Delayed diagnosis in sarcoma may have an impact on the patient’s opportunity for fertility preservation, psychological distress, patient dissatisfaction, and poor adherence to treatment [10, 11]. An Australian study (*n* = 91) found that patients with a delayed diagnosis for extremity sarcoma experienced distress relating to their diagnosis a year after treatment [12].

### The diagnosis interval for sarcoma

Sarcoma can be challenging to diagnose compared to other cancers due to its heterogeneous nature and rarity [13, 14]. Brouns et al. [15] reviewed patients recently diagnosed with soft tissue sarcoma and found that 47% of patients waited longer than a month before consulting a health professional, and 27% of patients took longer than a month to receive a diagnosis after consulting a health professional. Another study assessing 545 patients with a suspected sarcoma found that the median interval for diagnosis was 176 days from when the patient noticed their symptoms [16]. Goedhart et al. [17] determined that the mean interval amongst high-grade bone sarcoma patients ranged from 160 delays for patients with Ewing sarcoma, to 688 days for patients with chondrosarcoma. Diagnosis intervals for sarcoma appear to vary based on the aggressiveness of the tumour, with more aggressive tumours having shorter intervals on average.

Patients most commonly seek medical care because they have pain, want to know what the symptoms mean, are urged to seek a medical opinion, have a consultation for something else, or noticing presence of swelling/lump [16]. Dyrop et al. [16] examined the association between sarcoma patients’ symptoms and their time to diagnosis. The largest interval occurred between the time the patients noticed their symptoms and consulted a health professional. The reasons for this delay have not been thoroughly explored, with studies attributing it to sarcoma presenting with vague or intermittent symptoms [18].

Increased intervals also occur when health professionals provide incorrect diagnoses and refer patients for unnecessary tests [13, 19]. General Practitioners (GPs) have a significant role in reducing delays by ensuring patients receive appropriate management and treatment referrals [17]. Sarcoma patients frequently have multiple GP consultations, with bone sarcoma having amongst the highest rates of GP consultations amongst rare cancers [14]. A Korean study found that patients with osteosarcoma were sent for inappropriate procedures and tests, and were diagnosed with benign bone tumours, fractures and infections rather than sarcoma [20]. Similar findings have been reported in the UK [21], the Netherlands [18] and Denmark [16]. Children are particularly susceptible to late referral as their symptoms are dismissed because the possibility of a malignancy is low [13, 21]. Specialist centres can also prolong the diagnosis interval if the pathologist does not know how to safely test for sarcoma [19].

Although sources of delay have been reported internationally, there is a need to explore the sources of delay in the local context. Contextual factors, such as the availability of health services and referral protocol can affect the timeliness of diagnosis. There is a paucity of published research reporting patients’ perspectives of their sarcoma diagnosis, with most studies quantifying the delay intervals for diagnosis and examining the clinical implications of delays [15–17]. Carer perspectives are also under explored, and they may provide a unique insight and experience of diagnosis, such as if they were a carer of a young child who was diagnosed. The health professional perspective is also important to consider, as patients often engage with a range of health professionals before receiving a diagnosis.

The aim of this study was to explore patients’, carers’, and health professionals’ perceived barriers to timely diagnosis and referral for treatment for sarcoma.

## Methods

### Study design

An exploratory qualitative research design drawing upon a social constructionist interpretive framework was used. This framework places an emphasis on the participants’ views and experiences. The researchers’ intent was to make sense of the meanings the participants constructed [22].

#### Theoretical framework

The model of pathways to treatment was chosen to represent the processes, events, and intervals that can occur prior to diagnosis [23]. The theory acknowledges that there may be many possible pathways and barriers, and that diagnosis is not always linear. There are three key intervals before diagnosis: the appraisal interval, help-seeking interval, and diagnostic interval. The appraisal interval refers to the time between detecting bodily changes and perceiving a reasons to discuss these symptoms with a health professional. The help-seeking interval refers to the time between perceiving a reason to discuss symptoms and first consulting with a health professional. The diagnostic interval refers to the time between seeing a health professional and receiving a formal diagnosis.

### Participants

Data were collected using semi-structured interviews with health professionals, patients, and carers. Demographic information for patients, carers, and health professionals was obtained through a demographic survey and is presented in Tables 1, 2 and 3 respectively. Socio-economic status (SES) was determined with the Australian Bureau of Statistics’ Index of Relative Socio-economic Advantage and Disadvantage (IRSAD). The IRSAD calculates the SES of residential postcodes based on the economic and social information reported in the national census. IRSAD scores in the bottom quartile were classified as low, scores in the middle two quartiles were classified as moderate, and scores in the top quartile were classified as high SES [24].

**Table 1:** Patient demographics (*n* = 22)

Characteristic	Number
*Age at diagnosis (years)*	
Mean	43 (SD = 18.53, Min = 15, Max = 78)
*Time since diagnosis (months)*	
Median	32 (SD = 20.07, Min = 5, Max = 74)
*Sex*	
Male	9
Female	13
*Location*	
Metropolitan	16
Rural	6
*Socio-economic status*	
Low	1
Moderate	8
High	13
*Tumour location*	
Head and neck	2
Upper extremities	3
Spine	1
Torso	1
Pelvis	4
Lower extremities	11
*Type*	
Soft tissue	15
Bone	6
Both	1
*Histology* (Missing = 1)	
Chondrosarcoma	2
Osteosarcoma	4
Ewings	2
Epithelioid	1
Synovial	2
Gynaecological	1
Fibroblastic	1
Chordoma	1
Leiomyosarcoma	1
Pleomorphic dermal	1
Liposarcoma	1
Undifferentiated pleomorphic sarcoma	3
Malignant peripheral nerve sheath tumour	1
*Surgery*	
Resection	13
Bone excision	1
Limb salvage	3
Amputation	4
None	1
*Additional treatment*	
Chemotherapy	10
Radiation therapy	10
Hormonal therapy	1
Targeted therapy	2

**Table 2:** Carer demographics (n = 17)

Characteristic	Number
*Age (years)* (Missing = 2)	
Mean	51 (SD = 11.26, Min = 22, Max = 66)
*Sex*	
Male	5
Female	12
*Socio-economic status* (Missing = 2)	
Low	0
Moderate	5
High	10
*Duration as a carer (months)* (Missing = 2)	
Average (range)	33 (Min = 2, Max = 96)
*Relationship to patient*	
Mother	9
Father	1
Wife or female partner	3
Husband or female partner	3
Brother	1
*Age of patient at diagnosis (years)*	
Mean	29 (SD = 22.24, Min = 2, Max = 67)
*Tumour location*	
Head and neck	2
Upper extremities	2
Torso	1
Pelvis	2
Lower extremities	10
*Type*	
Bone	9
Soft tissue	7
Both	1
*Histology of patient* (Missing = 2)	
Osteosarcoma	5
Ewings	4
Epithelioid	1
Synovial	1
Gynaecological	1
Chordoma	1
Rhabdomyosarcoma	1
Chondrosarcoma	1

**Table 3:** Health professional demographics (n = 21)

Characteristic	Number
*Age (years)* (Missing = 1)	
Mean	44 (SD = 9.12, Min = 31, Max = 62)
*Sex*	
Male	10
Female	11
*Years of practice in current position* (Missing = 1)	
Average (range)	9 (SD = 7.75, Min = 1, Max = 27)
*Frequency working with sarcoma*	
Always	9
Often	7
Seldom	5
*Treatment/ management*	
Orthopaedic surgeon	3
General surgeon	1
Plastic surgeon	1
Medical oncologist	2
Radiation oncologist	1
Paediatric oncologist	1
Oncology ward nurse	1
General practitioner	1
Cancer nurse coordinator	2
Clinical nurse consultant	2
Clinical nurse specialist	1
Pathologist	1
*Post treatment/ ancillary*	
Youth counsellor	1
Youth worker	2
Exercise physiologist	1

#### Patients and carers

Eligibility criteria for patients included: over 16 years of age; diagnosed with sarcoma within the last 15 years; and able to converse freely in English. Family carers of patients were also invited to participate. Patient and carer participants were identified as eligible for the study by nurses and treating clinicians through a tertiary teaching hospital in a metropolitan setting in Western Australia. Recruitment was open to patients and carers living interstate. Interested participants were contacted via email and invited to participate. Patients and carers were also able to identify themselves for potential interviews through the Abby Basson Sarcoma Foundation (Sock it Sarcoma!). 22 patients and 17 carers were interviewed (27 face-to-face interviews and 12 phone interviews). Mean interview duration was 54 min (SD = 18 min, Min = 18 min, Max = 102 min).

#### Health professionals

Recruitment flyers were distributed through the specialist sarcoma centre. Snowball sampling and word of mouth were used to recruit additional health professionals currently working with sarcoma patients. 21 health professionals were interviewed (16 face-to-face, four phone interviews, and one electronic interview). Mean interview duration was 33 min (SD = 16 min, Min = 12 min, Max = 87 min).

### Data collection

An interview guide and prompts were used to elicit examples and to enable participants to elaborate on their answers. The interview guide was reviewed by previous patients and carers prior to study commencement, and contained questions about the participants’ perception of diagnosis (see Additional file 1). Participants were invited to be interviewed face-to-face in a mutually convenient location (e.g., their home), however some participants opted for a phone interview. This was due to the participant living interstate or rurally, or if their schedule did not permit them to meet face-to-face. One health professional from Perth opted to respond to the interview questions in a digital document, as they were not available for a verbal interview and were overseas throughout the data collection period. The interviews were undertaken by members of the research team (RW and GH), both of whom had experience conducting research interviews. Interviews were conducted between 10/05/2018 and 02/05/2019.

### Data analysis

Interviews were audio recorded and transcribed verbatim and analysed via thematic analysis. Data were managed with NVivo Version 12. Participant IDs were coded as follows patients – P, carers – C, and health professionals - HP. Patients’, carers’ and health professionals’ interviews were analysed at the same time to form an overall understanding of potential delays in diagnosis and individual perspectives about these delays. Braun and Clarke’s^26^ six phases for inductive thematic analysis were used to derive the themes. These phases include: familiarising with the data, generating initial codes, searching for themes, reviewing themes, defining and naming themes, and producing the final manuscript. Thematic analysis involves developing codes to represent data relevant to the research question. Similar codes are consolidated into themes and written up. This is an iterative process, meaning the researchers move back and forth between the different phases, in order to develop themes that best respond to the research question. Thematic analysis enables more rigorous data analysis and emphasis is given to the participant’s perceptions, feelings and experiences [25].

### Data saturation

Data saturation occurs when no new relevant information is obtained from participants [26]. Data were analysed concurrently with interviews after the first interview was conducted. When no new codes were generated during data analysis of the interviews, an additional interview was conducted to verify data saturation. Data saturation was conducted separately for each participant group. Recruitment continued until data saturation was reached for each participant group.

#### Quality and rigour

The study was reported according to the consolidated criterion for reporting qualitative research checklist (COREQ) [27]. Data coding was completed by two researchers (RW and GH) to identify different meanings and verify themes. An audit trail of all the study steps and decisions was kept.

### Findings

**Fig. 1:**
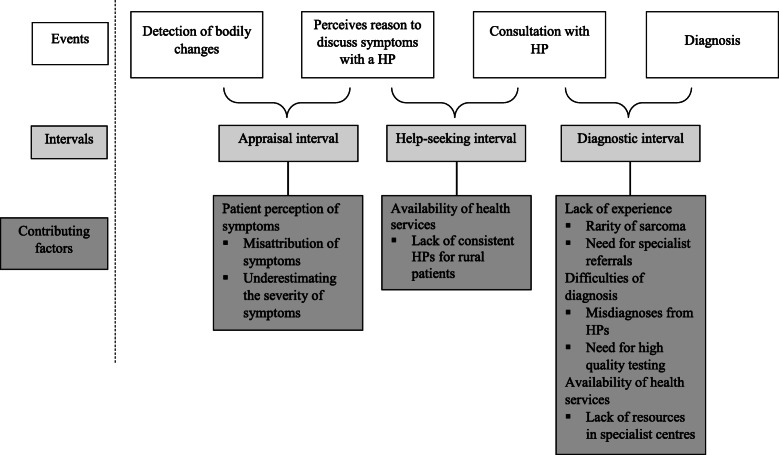
Model of intervals prior to diagnosis

Four overarching themes were identified: patient perception of symptoms, difficulties of diagnosis, lack of experience, and availability of health services. The intervals leading up to a sarcoma diagnosis are presented according to the model of pathways to treatment [23] (see Fig. 1). The events indicate where each interval starts and ends. Although the intervals are presented consecutively, patients may move back and forth between intervals. The contributing factors each influence the diagnosis interval, and are drawn from the key findings presented in the themes below.

### Patient perception of symptoms

This theme describes the patients’ perception of symptoms and how this may shape their decision to consult a health professional. This relates to the appraisal interval outlined in Fig. 1. Patients may misattribute their symptoms or underestimate the severity of their symptoms. For example, patients attributed their bodily changes to their lifestyle:

*“I was doing snowboarding at the time, and I thought I’d done something a bit weird, but it didn’t go away. “ P06*

Some patients may not perceive their symptoms as urgent and wait until they become too noticeable to ignore:

*“Patients often sit on a lump or an ache or a pain for frequently six to nine months… So they’re aware of a lump but it is painless so it’s ignored.” HP16*

*“It was something that we probably should have investigated earlier, but eventually it was very prominent so it was too large to ignore, so then that prompted us to go to the GP.” P04*

The site of the tumour and age of the patient may also shape the perceived need to consult a health professional. Participants reported that young patients more readily dismiss the severity of their symptoms or avoid presenting their symptoms because they are uncomfortable with its location:

*“It very much depends on where it is. Sarcoma in a long bone in a teenager, may be delayed in its presentation to us for a couple of months... ....A teenage boy with a sarcoma of the testes is not going to readily declare to his mother that he has a swollen testes and so we will often see paratesticular rhabdosarcoma quite advanced in the teenage group.” HP06*

A health professional suggested that further education for the community is needed to improve earlier symptom recognition and to prompt early medical consultation:

*“Also making it aware in the community that if they’ve got weird lumps and bumps that aren’t going away and, you know, a heat pack isn’t sort of fixing it” HP03*

### Lack of experience

Participants explained that the diagnostic interval may also increase when health professionals do not have sufficient experience with sarcoma (see Fig. 1). Underpinning this theme was the rarity of sarcoma, as participants observed that the health professionals who do not specialise in sarcoma may never see one in practice:

*“I’m not sure given the lack of frequency that a GP will ever see a sarcoma. I mean most of them will go through their career and never see a sarcoma.” HP02*

Participants also reported that rarer histologies may have particularly increased intervals:

*“They’re all rare, but there are some really, really rare sub-types … it took nearly four months to get a proper diagnosis because they just couldn’t find anybody else with this kind of sarcoma.” C06*

These intervals may occur when primary health professionals only refer for sarcoma testing after the more common possibilities are ruled out:

*“We go through routine investigations for other symptoms and eventually the sarcoma is picked up.” HP05*

Specialists may also contribute to delays if they are unfamiliar testing for sarcoma. A health professional suggested that patients may not be diagnosed if they are not referred to specialists who have regular contact with sarcoma. This was supported by a patient who was referred to the gynaecological team rather than the sarcoma service:

*“You’ve got to refer to the right specialist because sarcomas are so rare … . you need people who are seeing enough of these on a yearly basis in order to have at least some expertise.” HP08*

*“There’s a gap between the sarcoma team and the gynae team. I should have gone straight to the sarcoma team. But the gynae held on to me.” P05*

### Difficulties of diagnosis

This theme illustrates the intervals arising from misdiagnoses by primary and specialist health professionals (see Fig. 1). Patients described an extended interval between consulting a health professional and being referred for specialist testing. During this time, patients saw various primary health professionals, often multiple times, before they were referred on. This was attributed to primary health professionals assessing sarcomas as benign or attributing symptoms to the patient’s lifestyle:

*“This was a very delayed diagnosis. I had knee pain my right knee for about 18 months before it was diagnosed and I went to three GPs, two or three physios, and I took myself to an Emergency Department.” P13*

*“All soft tissue lumps are presumed by GPs to be lipomas so they’re always assumed to be lipomas and patients are falsely reassured.” HP16*

Intervals still occurred even after patients were referred for specialist testing. Some patients had several scans and biopsies that falsely reported their sarcoma as benign:

*“I had a swipe around in the first place, nothing there. I had a look-see and a curette, there was nothing there. I had multiple scans, CTs, nothing showed except for just a growth that is supposed to be benign.” P05*

The challenges in receiving a clear diagnosis was supported by a health professional who highlighted the importance of high quality testing:

*“The only delays now are in diagnostic challenges, if the pathologists can’t get enough tissue to be really clear about the diagnosis” HP02*

The technique used to complete the biopsy was also essential:

*“He said they would never have found out what they need to look for by doing that because they’d effectively sort of taken a skin shaving … ” P07*

Misdiagnosis was considered problematic as patients may go on to receive incorrect treatments, exacerbating the diagnosis interval. This may also lead to further recurrences and poorer outcomes:

*“The doctor at the time told me it was not cancer, it was easy to get rid of and that he would just operate, take it out and everything would be fine … Unfortunately he may have been an expert on tumours, but he wasn’t an expert on sarcoma tumours and he didn’t take wide enough margins which caused all the reoccurrences.” P21*

### Availability of health services

This theme refers to the impact of the limited availability of health services. The lack of resources was discussed when patients were referred to a specialist centre for testing or lived in areas with a paucity of health services. Rural patients reported difficulties in locating a primary health professional for consultation. This relates to the help-seeking interval (see Fig. 1), as patients who decided to seek medical advice were unable to readily consult a health professional. Some patients explained that there were limited primary healthcare options in their area:

*“It was probably the first time I could get in with a doctor because I'm in a rural area and it’s a little bit harder to get into the doctors.” P17*

The rural participants also discussed having poor continuity of care as local health professionals were only there for short intervals. There was also concern that the paucity of rural health professionals limited their ability to discuss and collaborate on cases:

*“Sometimes doctors stay for a couple of weeks and then bugger off again and then you’ll get a different one turn up and they all have their own agenda.” C12*

*“The thing with rural areas – you tend to be in smaller groups and more in isolation, and if there was the opportunity to discuss cases, people don’t do it in their practices anyway.” C07*

The lack of available resources was identified in specialist centres providing diagnoses. Participants reported increased intervals when specialist services lack the resources to provide timely testing:

*“Once they get to ‘the system’, they are occasionally delays because of the lack of manpower.” HP16*

*“We found out that it had been put on a pile waiting for someone to go and get the old scans so the specialist could compare them to them, and it just stayed on that pile.” C07*

## Discussion

This study aimed to explore patients’, carers’, and health professionals’ perceived barriers to timely diagnosis and referral for treatment. The study was informed by the model of pathways to treatment and the findings were mapped onto the intervals leading up to diagnosis (see Fig. 1) [23]. The findings raise an additional event that is not included in this model; the point when patients were referred to a specialist centre. Before referral, patients described being in a loop with primary health professionals who had limited experience with sarcoma. Referral to a specialist centre indicated that progress had been made towards diagnosis, however, delays still occurred due to the centre’s lack of resources and the need for high quality testing. The markedly different nature of these intervals suggests there may be scope to differentiate the diagnostic interval further, to include the point when patients receive a referral to a specialist centre.

### Strengths and limitations

The main strength of this study is the triangulation of data cross a variety of participants, enabling discussion of different barriers emerging at different time points of the diagnosis. The inductive study design facilitated the exploration of barriers, contributing to a better understanding of the high diagnosis interval. The study also has several potential limitations. One limitation is the inclusion of only a single GP participant. Two additional GPs were approached during recruitment; however, they were either not interested in participating or did not have the time to participate. This study was focused on recruiting health professionals through the tertiary hospital setting. Interviewing GPs may provide further explanation of barriers to diagnosing sarcoma, such as the high instance of multiple GP consultations [14]. Future research that focuses on understanding GPs’ perspectives on diagnosing sarcoma is required.

Patients were eligible to participate if they had been diagnosed in the last 15 years, with the median time since diagnosis being 2 years and 7 months (see Table 1). Some participants may have forgotten details or may have inadvertently misrepresented aspects of their diagnosis. Additionally, many patient and carer participants self-nominated to participate through the Sock it to Sarcoma Foundation, which may have reduced the variability of experiences across participants. This may have also contributed to the relatively high SES across the patient and carer participants (see Tables 1 and 2). To minimise this, we sought out additional referrals from the tertiary teaching hospital to reflect experiences of participants from different backgrounds. Some patients were recruited relatively soon after diagnosis (i.e., 2 months), and participants were recruited across differing sarcoma types (bone and soft tissue), histologies, ages, genders, and treatments.

### Context with other literature

Previous international studies have emphasised that sarcoma is prone to prolonged diagnosis intervals, which has been linked to its rarity, intermittent and varying symptoms, and frequency of inappropriate referrals [16, 28]. The current findings offer further insight into the reasons for the high interval. Patients perceived their symptoms to be lifestyle related, supporting findings by Miedema and Easley [21], who determined that cancer patients often attribute symptoms to exercise or strenuous work. The findings also suggest that young patients may more readily dismiss their own symptoms and have their symptoms misattributed by health professionals. Previous research has demonstrated that there is considerable difficulty in diagnosing cancer in adolescents and young adults, with sarcoma having amongst the highest rates of multiple GP consultations [29]. There is also a tendency for young people to perceive noticeable bodily changes as non-urgent [30]. Adolescents and young adult participants were wary about sharing their symptoms with their parents, which could reflect a tension between developing their independence and relying on their parents [21, 31].

Increased intervals also occurred after patients consulted health professionals. Several participants were incorrectly assessed by their GPs, which has been found to be the most frequent delay in soft tissue sarcomas [15]. Incorrect assessments often lead to inappropriate referrals and investigations. While it is important that patients are referred to specialists [32], the findings emphasise that patients with a suspected sarcoma need to be referred to a specialist who has experience with sarcoma. Beginning with a comprehensive history report followed by a rigorous clinical investigation may improve the frequency of appropriate referrals [15]. Diagnosis was also delayed due to the diagnostic challenges associated with sarcoma. The large number of potential sarcomas may increase the likelihood of misdiagnosis despite improvements in the quality of testing [33].

It is well documented that there is a shortage and poor retention of health professionals in rural areas [34, 35]. There is a paucity of research comparing outcomes between urban and rural sarcoma patients. Research examining outcomes for Ewings sarcoma patients found no difference in survival rates between urban and rural patients [36]. The present findings suggest that there may be inequalities for rural sarcoma patients during diagnosis, as the participants reported difficulty accessing and maintaining a primary health professional to manage their diagnosis.

### Clinical implications

Many countries, including Australia, have published referral guidelines for a suspected sarcoma. However, not every patient will experience all or any of the symptoms listed in the referral guidelines [37]. This presents a challenge to primary health professionals, as it may be unclear whether a patient should be referred to a sarcoma specialist unit for investigation. Although current recommendations suggest that patients should be referred through centralised pathways [38], the findings highlight that GPs may not be readily referring to centralised pathways. These specialist centres may also suffer from limited resources. Multispecialty diagnostic services are being trialled internationality to integrate specialist services to reduce the volume of patients that would otherwise be referred to the wrong specialists [39]. Developing multispecialty diagnostic services in Western Australia may support a centralised referral pathway and provide more opportunities for patients to access accurate diagnoses.

Reducing the appraisal intervals is difficult because of the limited incidence of the disease and the lack of public awareness of sarcoma [17]. Participants reflected that they would have seen a health professional sooner if they had known more about sarcoma, indicating that public health initiatives are needed to improve knowledge and early identification of sarcoma. It has been suggested that recent mass media campaigns in Australia have been successful in raising awareness leading to earlier detection of cancers [40]. A similar approach could be adopted with sarcoma, with media releases outlining common symptoms of sarcoma and encouraging early medical consultation.

### Policy implications

Clinical practice guidelines for referring a suspected sarcoma and how to progress to clinical review are available in Australia [41]. However, these guidelines are not being followed in all instances and there may be little awareness of the appropriate protocols. Future research could explore reasons for non-adherence to guidelines and interventions to support timely referral. At the systemic level, these guidelines and protocols need to be disseminated widely, particularly in primary care settings. Sarcoma education must be integrated into medical curricula and community awareness campaigns such as Sarcoma Awareness Month need to be further implemented nationally.

## Conclusions

Patients showing symptoms that could potentially be linked to sarcoma need to be promptly referred directly to a sarcoma specialist centre who can manage diagnostic testing and pathology and proceed to treatment as required. Developing multispecialty diagnostic services may improve access to specialist testing and reduce the dependence on centralised pathways. Increased education about sarcoma needs to be embedded into medical courses and professional development curricula to improve timely specialist referral. A public health approach to improve sarcoma knowledge and health seeking behaviours may reduce the time taken to consult a health professional.

## Supplementary information

**Additional file 1.**

## Data Availability

The interview data analysed in the study are not publically available to protect patient confidentiality, but are available from the corresponding author upon reasonable request.

## Abbreviations

C: Carer ID
COREQ: Consolidated criterion for reporting qualitative research checklist (COREQ)
GPs: General Practitioners
HP: Health Professional ID
IRSAD: Index of Relative Socio-economic Advantage and Disadvantage (IRSAD).
P: Patient ID
SES: Socio-economic status
