# Supplementary material for: The complexity of diagnosing sarcoma in a timely manner: perspectives of health professionals, patients, and carers in Australia
Source: BMC Health Serv Res. 2020 Aug 3;20:711. doi: 10.1186/s12913-020-05532-8 (PMC7397572; doi:10.1186/s12913-020-05532-8)
Supplement: Supplementary file 1 — Additional file 1. [file 12913_2020_5532_MOESM1_ESM.docx]

**Additional file 1**

**Interview guide for patients diagnosed with sarcoma**

1. What reasons/ symptoms prompted you to initially present to a health professional?
   1. How long after noticing these symptoms did you decide to see a HP?
   2. What prompted you to see a HP after waiting?
   3. How did you feel presenting your symptoms?
2. What was the route to diagnosis?
   1. What tests did they conduct to make a diagnosis?
   2. How long did you have to wait for your results?
   3. How did you feel waiting for your results?
   4. Did you feel like there were any delays to your diagnosis?
   5. What may have enabled a more timely diagnosis?
3. How did your clinician present your diagnosis?
   1. How did you feel receiving your diagnosis?
   2. How did receiving a diagnosis impact you?
   3. What did you know about sarcoma at the time of diagnosis?
   4. What kind of information about sarcoma did they provide you?
4. Did you feel like you had any unmet needs surrounding your diagnosis such as lack of information or support?
   1. Did you seek out any information independently?
   2. Did you have any difficulties understanding the information presented?
5. What was the timeframe between being diagnosed and receiving treatment?
   1. What may have enabled a timely treatment?
   2. What may have been a barrier to receiving treatment?
   3. Do you have any comments about the treatment that was prescribed to you?

**Interview questions for health professionals who are involved in diagnosing sarcoma?**

1. What is the typical timeframe between a patient’s first visit and a referral for further investigation at hospitals?
   1. What are the delays/challenges/barriers you think patients face in receiving a sarcoma diagnosis?
   2. Why do these delays occur? (e.g., patient, GP, system related?)
2. What are the delays/challenges/barriers patients face in receiving a treatment for sarcoma?
   1. Why do these delays occur? (e.g., patient, GP, system related?)
   2. How do you think this impacts on the individual?
3. What could mitigate or reduce these barriers to allow for a more timely diagnosis?
